# Laboratory Directions for Beginners in Bacteriology

**Published:** 1901-02

**Authors:** 


LABORATORY DIRECTIONS FOR BEGINNERS IN BACTERIOLOGY. An Introduction to Practical Bacteriology for Students and Practitioners of Comparative and Human Medicine. By VERANUS A. MOORE, B. S., M. D., Professor of Comparative Pathology and Bacteriology, New York State Veterinary College, and of Bacteriology, Cornell University Medical College, Ithaca, N. Y. Duodecimo, pp. 159. Illustrated. Second edition, enlarged and revised. Boston: Ginn & Co., 1900. [Price, $1.05]

Professor Moore has given his students a most compact and valuable little work on this subject, and so successful has it been in filling the gap that other teachers have adopted it in their laboratories. There are many excellent treatises on bacteriology, but very few which a student beginning the subject can use, hence the popularity which Professor Moore's little work has achieved.

One feature about all the books published by the members of the Cornell University faculty, is that nothing is left unsaid which the student is supposed to know. The minutest details are explained to the beginner and hence thoroughness is expected and demanded as he advances in the science. The idea is fully carried out in the outline of bacteriology and no one can go through its pages without becoming acquainted with the technique and the essentials of bacteriological investigation. No doubt the primer is but the beginning of a comprehensive work on the subject, and no one is better qualified to develop such a work than Professor Moore.

